# Human Papillomavirus *E6*/*E7* Expression in Preeclampsia-Affected Placentae

**DOI:** 10.3390/pathogens9030239

**Published:** 2020-03-23

**Authors:** Ashley L. Reily-Bell, Amanda Fisher, Bryony Harrison, Sara Bowie, Sankalita Ray, Mary Hawkes, Lyn M. Wise, Ryuji Fukuzawa, Erin C. Macaulay, Celia J. Devenish, Noelyn A. Hung, Tania L. Slatter

**Affiliations:** 1 Department of Pathology, Dunedin School of Medicine, University of Otago, Dunedin 9016, New Zealand; ashley.reily-bell@otago.ac.nz (A.L.R.-B.); amanda.fisher@otago.ac.nz (A.F.); bryony.nichole.harrison@gmail.com (B.H.); sara.bowie29@gmail.com (S.B.); sankalita.ray@postgrad.otago.co.nz (S.R.); tiritirimatangi0@gmail.com (M.H.); ryuji.fukuzawa@otago.ac.nz (R.F.); erin.macaulay@otago.ac.nz (E.C.M.); noelyn.hung@otago.ac.nz (N.A.H.); 2 Department of Pharmacology and Toxicology, School of Biomedical Sciences, University of Otago, Dunedin 9016, New Zealand; lyn.wise@otago.ac.nz; 3 Department of Pathology, School of Medicine, International University of Health and Welfare, Narita 286-8686, Japan; 4 Department of Women’s and Children’s Health, Dunedin School of Medicine, University of Otago, Dunedin 9016, New Zealand; celia.devenish@otago.ac.nz; 5 Maurice Wilkins Centre for Molecular Biodiscovery, Auckland 1142, New Zealand

**Keywords:** HPV, E6/E7, preeclampsia, placenta, trophoblast, RNAscope

## Abstract

Whether HPV is causative of pregnancy complications is uncertain. E6 and E7 affect functions underling preeclampsia (PET) in cultured trophoblasts, but whether E6 and E7 is produced in the placenta is uncertain. Here, we investigated whether *E6*/*E7* was expressed in the placentae from pregnancies with PET, other pregnancy complications (fetal growth restriction (FGR) and diabetes mellitus), and uncomplicated pregnancies. Placental tissues collected from two geographical locations were subjected to RNAscope analyses of high- and low- risk *E6/E7*, and individual HPV types identified using an HPV array. High-risk *E6/E7* expression was increased in both PET cohorts, (81% and 86% of patients positive for high-risk HPV DNA compared to 13% of control patients). Various HPV types were identified. Although HPV 18 was the most frequent in all cohorts, the majority of individuals had multiple HPV types (55% of the PET compared to 25% of the control cohort). Further evidence that E6 and E7 is present early when placental pathology underlying preeclampsia is established, is provided with the finding of high-risk *E6/E7* in the first-trimester placenta anchoring trophoblast. In conclusion, *E6/E7* expression and multiple HPV types were frequent in placentae from preeclampsia-complicated pregnancies.

## 1. Introduction

Human papillomavirus (HPV) is a prevalent infection amongst women of reproductive age [1]. During pregnancy, HPV infection rates are higher [1,2] and are associated with early and late-pregnancy complications, including PET, fetal growth restriction (FGR), and miscarriages [3,4,5,6,7,8,9,10,11,12,13,14,15,16]. However, an association between HPV and pregnancy complications is not found universally, and whether HPV is causative of pregnancy complications remains uncertain. The evidence supporting a pathological role of HPV in the placenta includes the identification of the HPV L1 capsid protein in regions with villitis, suggesting that HPV could be a factor in villitis cases with currently unknown etiology [6]. Other evidence also suggests an inflammatory modulatory function of HPV. Trophoblast cells engineered with HPV displayed increased expression of interferon-induced antiviral response genes [17,18] and activation of inflammatory regulators [19]. High-risk E6 and E7 proteins have well-characterized roles in cell transformation in a cancer context, but they may also affect placental function. HPV E6 and E7 proteins when introduced into trophoblasts reduced cell invasion, migration, and survival and poorly adhered to endothelial cells suggesting high-risk HPV affects functions underpinning an increased risk of PET [8,19,20].

For HPV to have a causative effect on placental cell function, HPV gene expression is required. Despite findings showing that E6 and E7 of high-risk HPV can affect key trophoblast functions in vitro, there is a lack of evidence of placental *E6/E7* expression. One investigation of HPV 16-positive placentae found no evidence that *E6/E7* was expressed [17]. Understanding the role of HPV in the placenta is complicated by multiple factors. Different estimates exist for HPV prevalence in the placentae, and the types present are largely uncharacterized [21]. Here, we determined the types of HPV and examined whether HPV *E6*/*E7* was expressed in placental trophoblasts using assays capable of detecting many HPV types in placentae from PET-associated pregnancies and first-trimester pregnancies to determine whether HPV gene expression was active in the placenta.

## 2. Results

### 2.1. High-Risk E6/E7 in Placentae from PET-Complicated Pregnancies

**Figure 1:**
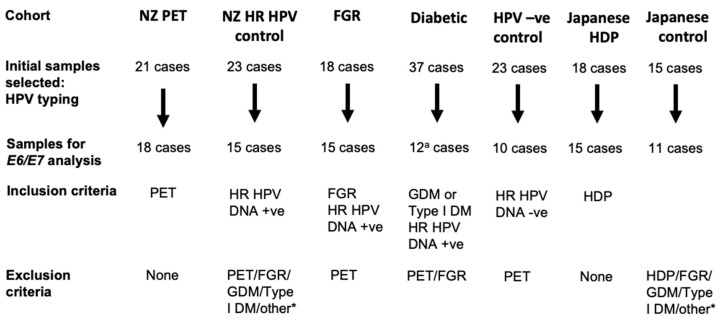
Cases selected for study. Cases with formalin-fixed paraffin-embedded material with complete clinical data were initially selected. The initially selected cases were tested for suitable RNA quality based on *ubiquitin C* (*UBC*) RNAscope were selected for the *E6/E7* RNAscope and HPV typing analyses. *, other complications, except for premature delivery and emergency C-section, were excluded. ^a^, not all initial cases were selected for the *UBC* control assay; PET, preeclampsia; FGR, fetal growth restriction; GDM, gestational diabetes; Type 1 DM, type I diabetes mellitus; HDP, hypertension disorders in pregnancy.

Whether *E6/E7* from high-risk HPV was expressed in HPV DNA-positive placentae, and whether *E6/E7* was more frequent in PET complicated pregnancies was investigated using RNAscope in five cohorts of placentae from New Zealand and two cohorts from Japan (Figure 1 and Table 1). Since high-risk HPV DNA was present in 100% of placentae from PET-complicated pregnancies in New Zealand in a previous study [6], in additional to a cohort consisting of placentae from pregnancies complicated with PET other cohorts were selected to determine if *E6/E7* was more likely in PET complicated pregnancies or any placentae with high-risk HPV DNA. Three additional cohorts (FGR, diabetes, and high-risk HPV control) contained cases selected based on a positive high-risk HPV DNA result from in situ hybridization, and included placentae with FGR or diabetes (both complications had a higher frequency of high-risk HPV DNA from a previous study), and a fourth cohort without any complications (high-risk HPV control) [6]. A fifth cohort contained cases tested negative for high-risk HPV DNA by in situ hybridization, as negative controls for the RNAscope analyses. The two cohorts from Japan were included to investigate the presence of *E6/E7* in placentae from a different geographical location and included a cohort of placentae complicated with hypertensive disorders in pregnancy (HDP) and a control cohort without complications. The HPV status of both Japanese cohorts was determined as part of this study. 

Before testing for *E6/E7* expression, sections from all placentae were tested for RNA quality using *UBC* as a positive control via RNAscope assay. This was performed as RNA quality can be compromised in the placenta due to difficulties in standardizing sampling and gene expression differences are affected by the delivery method and term of labor [22,23,24]. Placental tissue sections classified as having good or moderate RNA quality (Figure S1 and Figure 1) were investigated for *E6/E7* expression (Table 1); those with poor RNA quality were excluded. In the New Zealand PET cohort, 18 placentae (86%) had good or moderate RNA quality; of these, 16 placentae were positive for high-risk HPV DNA. In the FGR, diabetes and high-risk HPV control cohort, 15 placentae (83%), 27 placentae (73%), and 15 placentae (65%) respectively, had good or moderate RNA quality. In the Japanese HDP cohort 15 placentae (83%), and in the Japanese control cohort 11 (73%), respectively, had good or moderate RNA quality.

**Figure 2:**
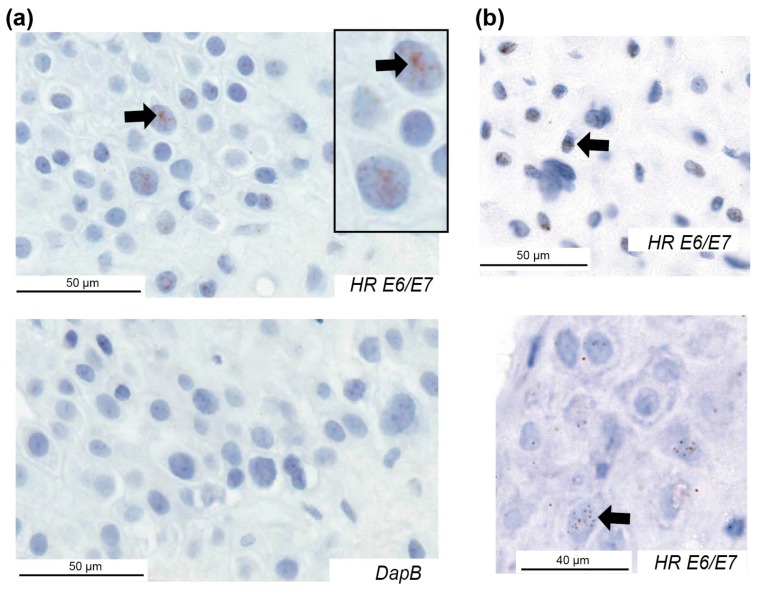
High-risk HPV *E6*/*E7* expression in the decidua basalis of placentae from pregnancies with preeclampsia. Examples of HPV *E6/E7* expression using an RNAscope assay for 18 high-risk HPV types in three cases and the negative control (*DapB*) for one case. Positive cells are indicated with arrows. HR, high-risk.

**Figure 3:**
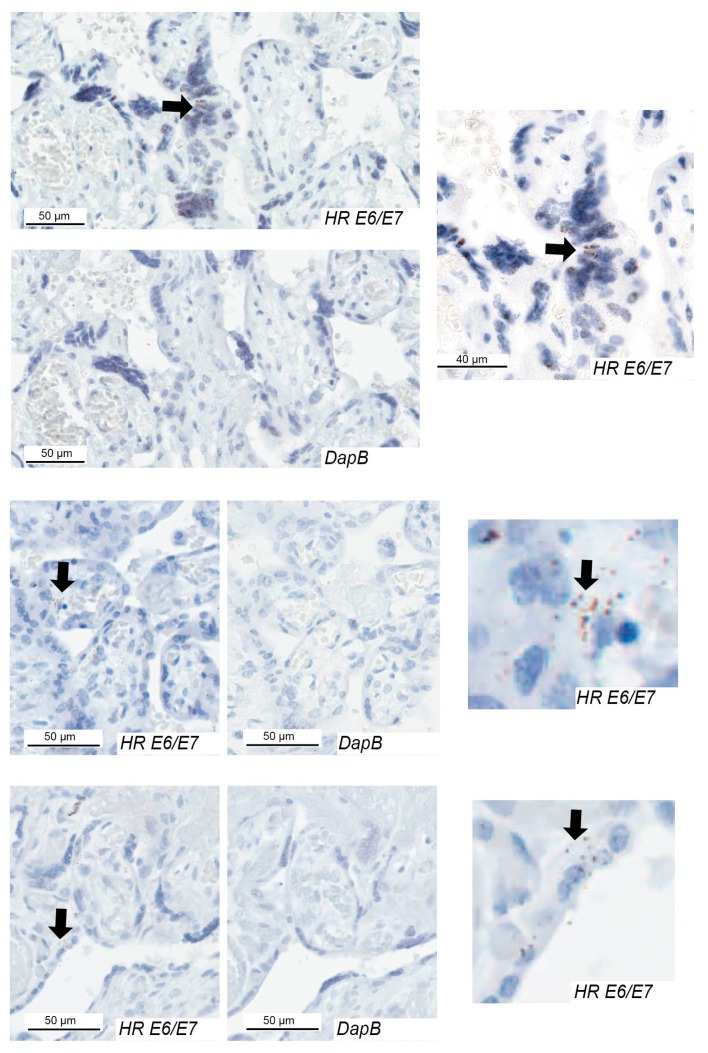
High-risk HPV *E6*/*E7* expression in the chorionic villus of placentae from pregnancies with preeclampsia. Examples of HPV *E6/E7* expression in three cases and the negative control (*DapB*) for each case. Positive cells are indicated with arrows. HR, high-risk.

Samples with good or moderate RNA quality were analysed for *E6/E7* using an RNAscope assay that can identify expression from 18 high-risk HPV types. The RNAscope assay showed strong positive staining in a positive control comprising HeLa cells (Figure S2C), and negative staining in the fifth cohort of placentae from New Zealand comprising ten placentae that were HPV DNA-negative with good or moderate RNA quality [6]. In the New Zealand PET cohort 81% of cases with high-risk HPV DNA showed *E6/E7* staining (Table 2). The majority of *E6/E7*-positive cases displayed positive staining in the decidua basalis (Figure 2), where 1–10% of cells were positive. In the chorionic villus (Figure 3), *E6/E7* expression was semi-quantified with a score between 0–3. Of the 13 PET cases positive for *E6/E7,* three had a score of 3, five had a score of 2, and five cases had a score of 1. The strongest *E6/E7* expression occurred within the syncytiotrophoblast, with some stromal cells positive. Some cases had positive dots in the nucleus only, cytoplasm only, or in both.

In the other cohorts, *E6/E7* was expressed in 40%, 25%, and 13% of placentae with high-risk HPV DNA from the cohorts with FGR, diabetes, and the HPV-positive control, respectively (Table 2). All cases had *E6/E7* expression in both the decidua basalis and the chorionic villus regions with the exception of one FGR case and one control where *E6/E7* was limited to the decidua basalis.

*E6/E7* expression was increased in the PET cohort compared with the HPV-positive control cohort (*p* = 0.0006). In the FGR and diabetic cohorts, *E6/E7* expression did not significantly differ from that of the HPV-positive control cohort. In the Japanese HDP cohort 6 out of 7 cases (86%) with high-risk HPV DNA had high-risk *E6/E7* expression (Table 3). None of control cases had high-risk *E6/E7* expression; however, one control had high-risk HPV DNA (*p* = 0.017 *E6/E7* HDP versus control group).

E6 degrades the p53 protein [25]. Consistent with *E6/E7* expression in the PET cohort, an analysis of cells positive for HPV (HPV L1 protein) and p53 found reduced p53 staining in HPV-positive cells in the decidua basalis of the PET cohort compared to the HPV-positive control cohort (Figure S3).

In summary, high-risk *E6/E7* was frequently expressed in placentae from PET-complicated pregnancies in a population with a high frequency of high-risk HPV DNA and in a second population where high-risk HPV DNA was less prevalent. 

### 2.2. Analysis of E6/E7 Expression with Low-Risk HPV

Low-risk HPV types are also found in the placenta [8,26]. Compared to high-risk HPV types, low-risk HPV types lack transforming properties and do not predispose patients to cancer, but they can affect cell proliferation and may be relevant to placental function [27]. To determine if *E6/E7* from low-risk HPV types may also be a factor in placentae from complicated pregnancies, an RNAscope assay of ten low-risk HPV types was performed with the New Zealand PET and HPV-positive control cohorts and the Japanese cohorts. In the New Zealand PET cohort 5 placentae (28%; Table 2) were positive for low-risk *E6/E7*, which all had high-risk *E6/E7* expression. Three placentae displayed low-risk *E6/E7* in the decidua basalis and chorionic villus, whereas such expression was limited to the decidua basalis in two placentae. Low-risk *E6/E7* was identified in one case in the high-risk HPV DNA-positive control cohort. *E6/E7* from low-risk HPV types was also present in PET-associated placentae but was not significantly increased compared to a control cohort without pregnancy complications. 

An analysis of low-risk *E6/E7* expression in the cohorts from Japan found five positive HDP cases (33%, Table 3). Two HDP cases with low-risk *E6/E7* were also positive for high-risk *E6/E7*. None of the control cohort had low-risk *E6*/*E7* expression. Low-risk *E6/E7* was significantly increased in the HDP cohort compared to the control group (*p* = 0.033).

In summary, low-risk *E6/E7* was also expressed in placentae from PET-complicated pregnancies. 

### 2.3. HPV Types in the PET Placenta

**Figure 4:**
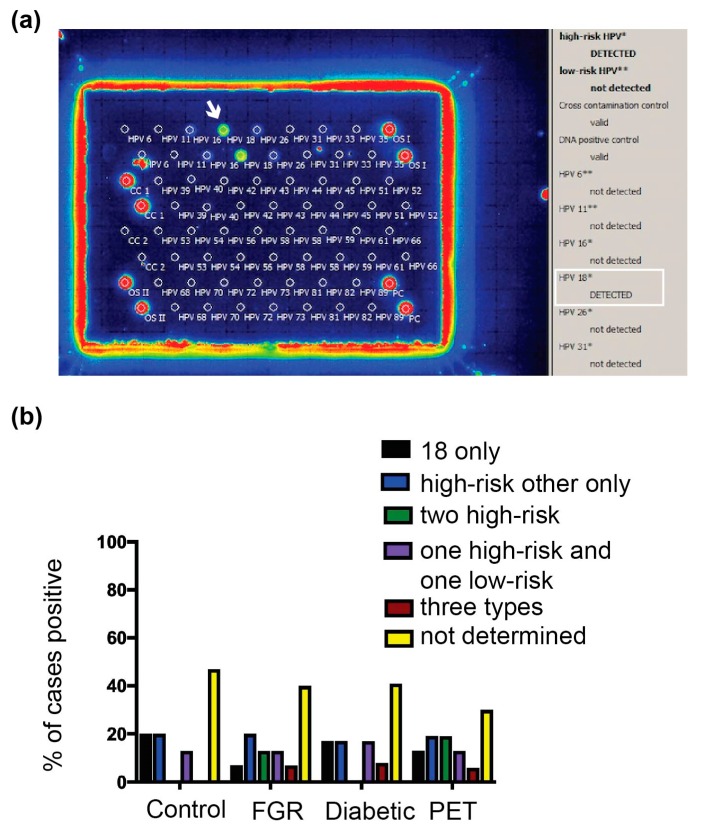
HPV types in the placentae. (**a**) Individual HPV types were identified using the EUROArray HPV test. The white arrow and white box highlight a placental sample positive for HPV 18. (**b**). HPV types identified amongst the New Zealand third-trimester placenta cohorts used in the *E6/E7* analyses. PET, preeclampsia; FGR, fetal growth restriction. The results are represented as the positive proportion for a given HPV type/s over the total number of cases with high-risk HPV DNA in the cohort. ND, not determined (the assay gave a negative result, most likely due to insufficient presence of HPV DNA to meet the Euroimmun HPV array assay criteria).

To determine if the increased *E6/E7* expression in the PET placentae was due to the presence of a different HPV type, individual HPV types were typed using a PCR and hybridization-based array method capable of detecting 30 HPV types (EUROArray HPV Test, Euroimmun, Lubeck, Germany). Individual HPV types were tested in the New Zealand cohorts used in the *E6/E7* analyses. The HPV typing array was successful in identifying the HPV type in most high-risk HPV DNA-positive cases identified by in situ hybridization (59%, n = 34/58; 53% of the HPV control, 50% of the diabetic, 60% of the FGR, and 69% of the PET cohorts; Figure 4).

HPV type 18 was the most frequently found type in the PET cohort (64% of cases with a type identified, n = 7/11). The presence of HPV 18 in the placenta was confirmed in five cases using an HPV 18-specific probe (Figure S2B and individual HPV types per case listed in Table S1). All cases had brown dots in cell nuclei, which were not present in five placentae determined as HPV negative by all testing methods, consistent with PET placentae containing HPV 18. HPV 18 was found at a similar frequency across the other cohorts. In the FGR, diabetic, and HPV-positive control cohorts (Figure 4), HPV 18 was present in 67% (n = 6/9), 50% (n = 3/6), and 63% (n = 5/8) of cases with an HPV type identified, respectively. In all cohorts, the next most frequent HPV type was 16, followed by other high-risk types 33, 35, 39, 45, 52, and 66. Low-risk HPV types (42, 55) and types of unclassified risk (26, 40, 53, 61, and 89) were also found amongst the cohorts. Across all cohorts, 25–56% of cases contained more than one HPV type, with the PET and FGR cohorts having the most and the HPV-positive control cohort having the fewest cases with multiple HPV types. Cases in the FGR, PET and HR HPV control cohorts were all tested with the Leica Bond probe towards five HPV types. Three PET cases and one FGR case only contained HPV types that were not covered by the probe so are likely to contain an additional HPV type. Therefore 82% of PET (n = 9/11) and 67% (n = 6/9) of FGR cases are likely to contain multiple HPV types (*p* = 0.024; PET versus the HR HPV control group).

Ten cases determined as HPV negative by in situ hybridization were included as controls; the more expansive HPV array typing method confirmed that all HPV-negative cases were negative [6]. There were no differences in HPV types amongst the cohorts; therefore, the increased *E6/E7* expression in the PET cohort was not due to the presence of a different HPV type. However, this study suggests the presence of multiple HPV types may be relevant to PET. 

### 2.4. HPV E6/E7 was Present in First-Trimester Placentae

If *E6/E7* were also expressed in early gestation when the foundations for PET are established, it would suggest an effect of *E6/E7* on PET. To determine if *E6/E7* was expressed in first-trimester placentae, 32 cases (n = 32/40, 80%) had sufficient decidua basalis and chorionic villus tissue and good or moderate RNA quality necessary to assess high-risk *E6/E7* expression. The same cases were also typed for the presence of high-risk HPV DNA using in situ hybridization. Consequently, 46% of cases were positive for high-risk HPV DNA; fourteen cases displayed HPV DNA in the decidua basalis tissue in both the endometrial epithelium and endometrial stromal cells/extravillous trophoblast, and seven cases were positive in the chorionic villi (six of which also had HPV DNA in the decidua basalis) (Figure S3 and Table 4). In endometrial epithelial cells, *E6/E7* staining was observed in 57% of cases with HPV DNA (n = 8/14). Minimal *E6/E7* was present in each cell; however, multiple cells were positive (Figure S4A). In chorionic villi, 43% (n = 3/7) of cases showed moderately intense staining for *E6/E7* in the cytotrophoblast and the syncytiotrophoblast (Figure S4B). 

**Figure 5:**
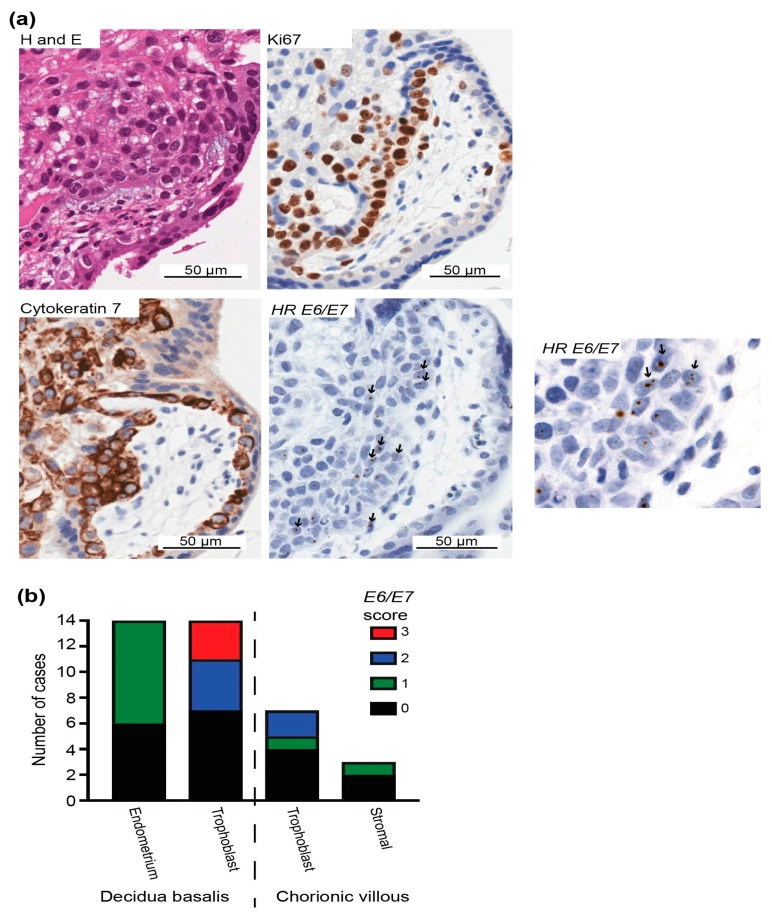
HPV *E6*/*E7* expression in the first-trimester placenta trophoblast. (**a**) H&E staining and immunohistochemistry for the epithelial marker cytokeratin 7 to identify trophoblasts with the most frequent *E6/E7* expression using the HPV-HR 18 RNAscope probe. *E6/E7*-expressing cells were evident in regions with proliferating (Ki67-positive) trophoblasts. Arrows highlight *E6/E7*-positive cells. (**b**) Evaluation of high-risk HPV E6/E7 expression in the first-trimester placenta. Early gene expression of HPV *E6/E7* in different cell types was scored as 0, 1, 2, or 3 based on the positive staining intensity and positive cell number to highlight the cell types with more *E6*/*E7*, as follows: 0, E6/E7 negative; 1, faintly positive dots in at least 6 cells or moderately intense positive dots in <3% of cells overall; 2, moderately intense positive dots in >3% and ≤20% of cells; and 3, moderately intense positive dots in >20% of cells.

In chorionic villi, 33% of stromal cells (n = 1/3 cases with HPV DNA) showed positive *E6/E7* expression. Cells with the most intense staining were identified as anchoring or extravillous trophoblasts based on the observation that areas with *E6/E7*-positive cells were also positive for Ki67 and cytokeratin, indicating proliferating cells and epithelial cells, respectively (Figure 5 and Figure S4B). In three cases, more than 20% of trophoblast nuclei were positive for *E6/E7.*

**Figure 6:**
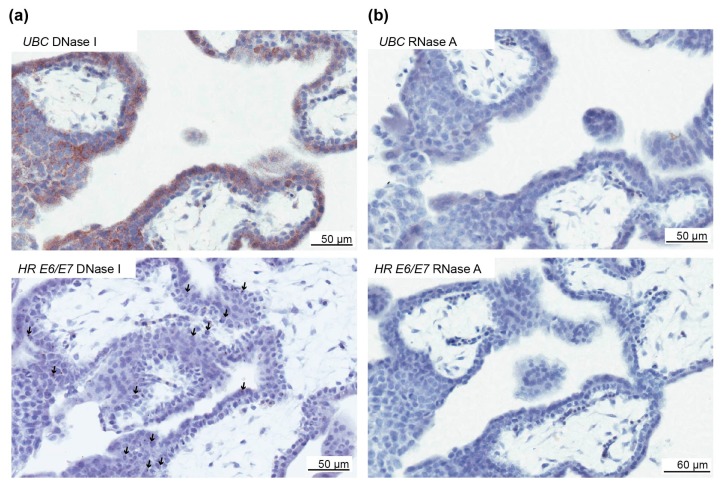
Validation of high-risk HPV *E6*/*E7* expression in the first-trimester trophoblast. To determine if the *E6*/*E7* RNAscope assay was detecting RNA and not DNA, first-trimester placental tissues were pretreated separately with DNase I and RNAse A. (**a**) Top panel, *Ubiquitin C* (*UBC*) staining following DNase treatment to illustrate the presence of RNA. Bottom panel, positive staining for high-risk (HR) E6/E7 with DNase I treatment to illustrate the presence of staining when HPV RNA is present. (**b**) Top panel, *UBC* staining following RNase A treatment to illustrate the absence of RNA. Bottom panel, Absence of *E6*/*E7* staining following RNase A treatment to illustrate the absence of staining when RNA is removed. The arrows highlight *E6/E7*-positive cells.

Human papillomavirus DNA can denature into single-stranded DNA, which could be detected by the RNAscope assay [28]. To validate whether HPV RNA was present, three cases with *E6*/*E7* in villous trophoblasts were separately pretreated with DNase I and RNase A before repeating the *E6*/*E7* RNAscope assay. *E6/E7* was present following pretreatment with DNase I, but not with RNase A, in all three cases (Figure 6), suggesting *E6/E7* RNA is present.

The three cases exhibiting high-risk *E6/E7* in the chorionic villi were investigated to determine whether low-risk *E6/E7* was also present; consequently, one case was found to be positive (Figure S2D). To test if the HPV was part of a productive infection *E4* expression was investigated using RNAscope and a probe that could detect *E4* from HPV 16 and 18. *E4* was present in 38% of cases (n = 3/8) of those positive for high-risk E6/E7. *E4* was present in the decidua basalis and no evidence of staining in the chorionic villus was found.

These results suggest that high-risk HPV genes associated with altered trophoblast function in vitro are expressed in the first-trimester placenta.

## 3. Discussion

The role of HPV as a pathogen likely extends beyond cancer promotion, as a growing number of studies have found associations between HPV and increased risks of pregnancy complications and cardiovascular diseases [29,30,31]. This study found active HPV gene expression in placentae in PET-complicated pregnancies, suggesting that HPV genes capable of affecting cell function are expressed in PET associated placentae. The explanation for increased HPV *E6/E7* RNA in PET-associated placentae is uncertain, but it is likely not due to the presence of different HPV types compared to placentae from uncomplicated pregnancies. 

In the current study, *E6/E7* was frequently expressed in HPV DNA-positive placentae from two cohorts of PET-complicated pregnancies, but it was rare in the placentae from uncomplicated pregnancies, including those with high-risk HPV DNA. This suggests that in PET cases, HPV oncoproteins affecting cell function likely occur in the placenta if high-risk HPV DNA is present [25,32]. In trophoblast cultures, the introduction of HPV influenced the resulting trophoblast phenotypes, which would impair implantation and placental maintenance that underpin PET [8,20,33,34,35]; this was also observed in a study that only introduced E6 and E7 without the whole HPV genome [20]. 

Few studies have investigated HPV DNA in the placenta and preeclampsia. Instead, most studies investigated the whole of cervical HPV in preeclampsia and reported varying associations that may relate to HPV prevalence in the study population [5,36]. In the study by McDonnold *et al.* (2014), cervical HPV during early pregnancy posed a two-fold increased risk of preeclampsia [5]. The finding of *E6*/*E7* expression in the anchoring trophoblast is consistent with *E6/E7* being active early in placentation, when HPV would likely have the strongest effects on PET based on the in vitro effects of *E6/E7* in trophoblasts.

Other consequences regarding placental function could occur when *E6/E7* is expressed. In addition to decreased p53 and RB function, E6 and E7 degrade and interact with many other proteins, including those with roles in inflammation, and both proteins are required for the long-term episomal maintenance of HPV [37,38,39]. 

Establishing that high-risk E6 and E7 in the placenta are causative of PET requires more investigation. High-risk E6 and E7 are found in women with normal cervical cytology, suggesting that HPV gene expression is not always associated with pathology [40,41]. Most placentae positive for high-risk HPV *E6/E7* had limited *E6/E7* expression overall, which was much lower than that found with integrated HPV, as illustrated in HeLa cells in the current study, or than the amount likely achieved in cultured trophoblasts transfected with E6 and E7 constructs. Episomal HPV is capable of affecting cell function, as evidenced by the existence of invasive cervical cancers with only an episomal virus, but these cancers were associated with high *E6* expression and a high viral load [42]. In this study, reduced p53 was found in HPV-positive cells in PET-associated placenta cases, consistent with the presence of E6 function, but additional studies are required to determine whether *E6/E7* is sufficient to affect overall placental function. If increased HPV activity resulted in increased placental cell apoptosis as found in vitro, cells with the highest *E6/E7* expression may not be represented in the in situ analysis, therefore limiting the use of in situ analyses to predict the consequences of HPV gene expression [8]. HPV is often found as a co-infection with other sexually transmitted infections [43]. Establishing HPV as a causative agent in PET will require an analysis of other sexually transmitted infections in the PET-associated placenta to establish if HPV alone, another infection, or combined infection is most deleterious towards placental function. 

HPV 16 *E6/E7* expression was not found in placentae using quantitative PCR in another study [17]. The results from the current study suggest that *E6/E7* is not always expressed in HPV-positive cases and may be more likely in PET compared to other pregnancy complications with HPV-positive placentae, which adds further complexities to deciphering how placental HPV *E6/E7* expression is governed. High-risk *E6/E7* was not significantly increased in placentae from FGR- and diabetes-complicated pregnancies; however, a considerable number of FGR placentae displayed *E6/E7* expression, and an association between the two would be better established in a larger cohort of placentae.

Low-risk *E6/E7* was present in over 20% of PET placentae from two geographical locations. In the Japanese cohort, low-risk HPV *E6/E7* was significantly increased compared to the control cohort. Whether low-risk HPV contributes to pregnancy complications in the absence of high-risk HPV is uncertain. Low-risk HPV types are not carcinogenic and instead cause benign proliferative lesions; thus, they are not considered a serious health concern. If high-risk HPV proves to be causative of PET, determining whether low-risk HPV is also of “low risk” for PET may be important. The function of low-risk E6 and E7 is less understood. Roles in maintaining viral episomes have been proposed [44], and recent evidence suggests that some functions of low-risk E6 are similar to those of high-risk E6. High- and low-risk HPV E6 degraded the Na^+^/H^+^ exchanger regulatory factor 1 protein, resulting in activation of the Wnt signaling pathway [45].

HPV 18 was the most frequent type in the PET, control, FGR and diabetic groups. Although there were no differences regarding individual HPV types in the PET cohort many PET and FGR cases contained multiple HPV types. The prevalence of multiple HPV types varies between populations. In cervical screening studies estimates of 4.6% and 43.2% of HPV positive women have multiple HPV types present [46,47]. Women with multiple HPV infections are at a higher risk of cervical disease, and multiple infections were common in women with few lifetime sex partners, suggesting multiple HPV infections are not only determined by increased sexual partners but also determined by other factors such as immunological regulation [47].

Whether HPV vaccination will reduce PET incidence is yet undetermined. Currently, the Gardasil 9 vaccine (Merck Sharp and Dohme, Kenilworth, NJ, USA), the FDA-approved HPV vaccine effective against the most types, would cover the HPV types most frequently found in this study; however fourteen cases had an HPV type, including three cases with high-risk types, that would not be vaccinated against by Gardasil 9. Pregnancies complicated by PET are associated with a predisposition to cardiovascular disease for both the mother and child. Therefore, understanding the underlying mechanism and role of HPV may provide novel insights for preventing cardiovascular disease [48,49].

## 4. Materials and Methods 

### 4.1. Sample Collection

Placental samples were collected from New Zealand and Japan. For the New Zealand samples, placentae were selected from those collected during the Otago Placenta Study (OPuS) based at Dunedin Hospital, including previously studied cases and newer cases (collected since 2014) [6]. The cohorts used in this study are outlined in Table 1, and the case selection criteria are summarized in Figure 1. Briefly, cases were selected based on the presence or absence of pregnancy complications, presence or absence of high-risk HPV DNA, availability of frozen and formalin-fixed paraffin-embedded (FFPE) material, and placental tissue with good or moderate RNA quality based on positive control staining using RNAscope with *ubiquitin C* (*UBC*; Figure S1). For the New Zealand samples, preeclampsia was clinically diagnosed according to the SOMANZ guidelines (https://somanz.org), and FGR was defined as weight ≤5th centile for gestational age, as determined by the GROW-centile calculator with permission from the Perinatal Institute (http://www.perinatal.org.uk). For the cohort from Japan, growth estimates were calculated according to the standard gestational age-specific birth weight set by the Japan Pediatric Society, and preeclampsia was defined according to the guidelines of the Japanese Society of Hypertension [50].

For New Zealand and Japanese participants: none of the women had been vaccinated against HPV. Cases without other infections were selected by clinical investigations during pregnancy; cervical swabs for *Chlamydia trachomatis* or *Neisseria gonorrhoeae* DNA; vaginal swab culture for bacterial vaginosis, fungi, or *Trichomonas vaginalis*; and maternal serology (HIV, syphilis, hepatitis BsAg, toxoplasmosis, and rubella). Cases with any doubt or suspicion of an organism other than HPV were excluded from the study. Clinical data were available concerning smoking status, medical history, maternal age, maternal BMI, and gestation. Ethnicity data was available for the New Zealand cohort (80% identified as New Zealand European, 10% as Maori, and 10% identified with other ethnicities or more than one ethnicity). 

To determine whether HPV *E6/E7* was expressed in early pregnancy, a cohort of 40 first-semester placentae were obtained from elective terminations (<12 weeks’ gestation) collected from 2015 to 2017 in New Zealand on a convenience basis. Thirty-two cases contained decidua basalis and chorionic villus tissue and had sufficient RNA quality, as determined using RNAscope for *UBC* (>6 dots per cell). 

Ethical approval was obtained from the New Zealand Health and Disabilities Lower Regional South and Multiregional Ethics Committees, and all participants provided signed informed consent. In Japan, ethical consent was obtained from the ethics committee of Tokyo Metropolitan Tama Medical Center.

### 4.2. HPV DNA in situ Hybridization

The presence of high-risk HPV DNA was determined either in a previous study [6] using a probe towards 13 high-risk types or in this study using an automated staining method to 5 high-risk types, as outlined below. All cases in the FGR and PET cohorts regardless of whether they had been typed in the previous study were also typed in this study using the probe towards 5 HPV types. FFPE sections intended to fill the width and breadth of a standard histology cassette were assayed with the Bond Ready-to-Use ISH HPV Probe (Leica Biosystems, Wetzlar, Germany), which detects five common high-risk HPV types (16, 18, 31, 33, and 51), and an automated staining method using the Leica Bond RX Research Autostainer (Leica Biosystems, Wetzlar, Germany). The staining protocol and reagents recommended by the manufacturer were used with minor modifications: the probe hybridization step was conducted for 3 h instead of 2 h, and a 3,3’-diaminobenzidine (DAB) enhancer step (Leica Biosystems, Wetzlar, Germany) was included. 

To confirm that some placentae had HPV 18, an HPV 18-specific probe (HPV Type 18 Probe, Digoxigenin, Biocare Medical, Pacheco, CA, USA) was used on selected cases identified as likely positive for HPV 18 based on HPV array analysis. Cell clots containing HPV18-positive HeLa cells were used as positive tissues. For in situ hybridization, tissues were treated with Carezyme III Protease (Biocare Medical, Pacheco, CA, USA) for 10 min at room temperature (20–26 °C) and heated in Tris-EDTA (pH 9) buffer at 95 °C for 20 min. Then, the probe was applied and denaturation occurred at 95 °C, followed by hybridization for 20 h at 40 °C. After hybridization, slides were placed in SSC wash buffer (Biocare Medica, Pacheco, CA, USA) at 40 °C for 5–10 min, and the probe was detected after applying anti-digoxigenin antibody (Biocare Medica, Pacheco, CA, USA), EnVision Dual Link (HRP Rabbit/Mouse, Dako, Glostrup, Denmark), DAB (Cell Marque, Rocklin, CA, USA), and DAB enhancer (Leica Biosystems, Wetzlar, Germany). Slides were counter-stained, dehydrated, and mounted following a standard procedure. 

Positive staining examples are shown in Figure S2A (probe towards five high-risk HPV types) and Figure S2B (probe towards HPV 18 only).

### 4.3. HPV Typing

Individual HPV types in placental tissues collected in New Zealand were identified using the EUROArray HPV Test (Euroimmun, Lubeck, Germany). DNA was extracted from frozen tissue using the QIAamp DNA Mini Kit (Qiagen, Hilden, Germany) according to the manufacturer’s protocol, which was modified by adding poly-A carrier RNA (Qiagen, Hilden, Germany) to improve DNA extraction. HPV typing was conducted following the manufacturer’s protocol with minor modifications: the hybridization step was increased from 40 min to 90 min, and in some cases, more than the recommended DNA quantity was added (>100 ng). 

### 4.4. RNAscope 

The RNAscope 2.5HD Assay-Brown manual assay protocol (Advanced Cell Diagnostics, Newark, CA, USA) was carried out on 5 µm FFPE sections using a protocol modified according to the manufacturer’s recommendations for placenta (the protease plus digestion step was decreased from 30 to 25 min). The Positive Control Probe Hs-UBC towards *UBC*, Negative Control Probe DapB towards the bacterial gene *DapB*, HPV-HR18 probe towards *E6/E7* of 18 high-risk HPV types (16, 18, 26, 31, 33, 35, 39, 45, 51, 52, 53, 56, 58, 59, 66, 68, 73, and 82), and LR-HPV-HPVE6/E7 probe towards 10 low- or unclassified HPV types (6, 11, 42, 43, 44, 54, 61, 70, 72, and 81; Advanced Cell Diagnostics, Newark, CA, USA) were applied to placental tissue slides and FFPE HeLa cell slides as a control for high-risk *E6/E7* expression (Figure S2C). The first trimester cohort positive for *E6/E7* were analysed for HPV *E4* expression using the V-HPV16/18-E4 probe towards *E4* of two high-risk types (16 and 18). Slides were counter-stained, dehydrated, and mounted following a standard procedure.

Since the E6/E7 RNAscope assay could detect single-stranded DNA, three first-trimester placenta tissue sections that were positive for the *E6*/*E7* assay were pretreated with RNase A (PureLink RNase A, Invitrogen, Carlsbad, CA, USA) for 30 min at 40 °C and DNase I (Ambion DNase 1 [RNase-free], Invitrogen, Carlsbad, CA, USA) separately for 30 min at 40 °C, and the *E6*/*E7* RNAscope assay was repeated.

### 4.5. Slide Analysis

Stained slides were scanned into the digital pathology scanners of the Aperio Scanscope CS or the Aperio VERSA System (Leica Biosystems, Wetzlar, Germany) at up to 630× magnification under oil. The entire tissue section was viewed to identify cells positive for each component. For the HPV DNA analysis, cases were classified as negative if <6 positive cells were present throughout the tissue section. For HPV RNAscope analyses, placentae were examined in different tissue compartments (decidua basalis endometrium, epithelium, stromal cells, and the chorionic villus including the cytotrophoblast and syncytiotrophoblast, and chorionic stromal cells). 

*Third-trimester placenta cohorts: E6/E7*-positive staining was scored based on positive stains in the decidua basalis and/or chorionic villus, the number of positive high-power fields (hpfs; ×400 magnification) in the chorionic villus, and the number of positive dots within chorionic villi cells. Stains were scored as 0 (no evidence of positive staining throughout the tissue section, including the decidua basalis and chorionic villus), 1 (either positive only in the decidua basalis, more than ten positive dots in at least 5–10 hpfs in the chorionic villus, or >20 and ≤50 positive dots in 2 hpfs), 2 (positive in both the decidua basalis and chorionic villus with either more than ten positive dots in >5 and ≤20 hpfs or 2 hpfs with >50 up to 200 positive dots), or 3 (positive in both the decidua basalis and chorionic villus with either more than ten positive dots in >20 hpfs or two hpfs with >200 positive dots).

*First-trimester placenta cohort*: *E6/E7* staining was scored as 0–3 in different cell types based on the positive staining intensity and positive cell number, which highlighted cell types with more *E6/E7*, as follows: 0, *E6/E7* negative; 1, positive dots in at least six cells or intensely positive dots in <3% of cells overall; 2, intensely positive dots in >3% and ≤20% of cells; and 3, moderately positive dots in >20% of cells.

Equivalent areas with positive staining on HPV-stained sections were checked on the *DapB-*stained slides to exclude non-specific staining.

### 4.6. Immunohistochemistry

Immunohistochemistry for cytokeratin 7 (clone RN7, Leica Biosystems, Wetzlar, Germany) and Ki67 (clone SP6, Thermo Fisher Scientific, Waltham, MA, USA) was conducted according to an automated staining method using the Leica Bond Polymer Refine Detection System and the Leica Bond RX Research Autostainer (Leica Biosystems, Wetzlar, Germany). A double-staining method for p53 (clone SP5, Cell Marque, Rocklin, CA, USA) and HPV L1 1 HPV-viral capsid protein (clone K1H8, Dako, Glostrup, Denmark) was performed according to an automated staining method with the same Leica system. HPV L1 and p53 were detected using the BOND polymer refine red detection kit and the BOND polymer refine detection (DAB) platform (Leica Biosystems, Wetzlar, Germany) through a sequential staining method.

Stained slides were scanned into the Aperio Scanscope CS digital pathology system (Leica Biosystems, Wetzlar, Germany) at up to 400× magnification. Slides were investigated manually to identify positive cells.

### 4.7. Statistical Analyses

χ^2^ tests and t-tests were used to compare differences between categorical measures and continuous measures, respectively. Statistical significance was indicated by a two-tailed *p*-value < 0.05. The Bonferroni correction used to correct for multiple comparisons. 

## 5. Conclusions

This study provides evidence that HPV *E6/E7* is expressed in the PET placenta. This result, combined with the observed *E6/E7* expression in the first-trimester trophoblast, suggests that HPV is active early in placental development when placental origins of preeclampsia are established. Further investigations are required to establish whether the *E6/E7* quantity expressed is sufficient to affect placental function and if HPV is causative towards pregnancy complications. 

## Figures and Tables

**Table 1:** Cohort characteristics used in the high-risk *E6/E7* analyses.

	New Zealand PET	New Zealand HR HPV Control	New Zealand FGR	New Zealand Diabetic	New Zealand HPV Negative	Japan HDP	Japan Control
	(n = 18)	(n = 15)	(n = 15)	(n = 12)	(n = 10)	(n = 15)	(n = 11)
**Maternal age (years)**	28.3 (5.8)	32.5 (7.2)	31.7 (2.7)	31.9 (5)	30.6 (9.3)	36.1 (4.4)	35 (6.2)
**Delivery method**							
*Emergency C section*	39%	20%	13%	8%	0%	47%	0%
*Elective C section*	11%	33%	47%	59%	20%	33%	9%
*Vaginal*	50%	47%	40%	33%	80%	20% ^a^	91%
**BMI**	27.7 (6.3)	25.8 (5.7)	27 (6.6)	29 (6.1)	23.4 (4.6)	23.4 (4.6)	21.5 (4.8)
**Smoking**	11%	27%	27%	25%	20%	7%	0%
**Gravida**	2 (1–4)	2 (1–4)	2.5 (1–4.25)	1.5 (1–3)	2 (0–2)	2 (0–2)	1 (0–1)
**Parity**	1 (0–1)	1 (0–1)	1 (0–2.5)	0.5 (0–1.75)	1 (0–2)	1 (0–2)	0 (0–1)
**Miscarriage**	0 (0–0)	0 (0–0)	0 (0–0)	0 (0–0.75)	0 (0–1)	0 (0–1)	0 (0–1)
**Baby sex** **(% female)**	55%	67%	40%	42%	50%	35%	64%
**Gestation weeks**	36.2 (3.4)	37.6 (3)	36.6 (2.8)	36.9 (1.9)	38.9 (1.8)	36.6 (1.7)	39.2 (1.3)
**Fetal growth measurement ^1^**	28.1 (27.8)	38.4 (28.7)	8.48 (14.9)	72.4 (35.7)	32.1 (28.3)	−0.97 (1.4)	−0.32 (0.52)
**High-risk HPV positive**	89% ^2^ (n = 16)	100% ^3^ (n = 15)	100% ^3^ (n = 15)	100% ^3^ (n = 12)	0% ^3^ (n = 0)	47% (n = 7)	9% (n = 1)

^1^, Personalized growth centile (NZ); gestational age-specific birth physique standard value (Japan). ^2^, Evidence from in situ hybridization or the HPV typing array results. ^3^, Samples selected based on the presence or absence of high-risk HPV DNA by in situ hybridization. BMI, body mass index; HR, high-risk, PET, preeclampsia; FGR, fetal growth restriction; HDP, hypertension disorders in pregnancy. The results represent the mean ± sd with the exception of gravida, parity, and miscarriage median (25th–75th percentile).

**Table 2:** High- and low-risk E6/E7 in third-trimester placentas from New Zealand positive for high-risk HPV DNA.

Cohort	PET (n = 18)	HR HPV Control (n = 15)	FGR (n = 15)	Diabetic (n = 12)
**High-risk HPV DNA positive**	89% (n = 16/18)	100% (n = 15/15)	100% (n = 15/15)	100% (n = 12/12)
**High-risk *E6/E7/* high-risk HPV DNA positive**	81% ^1^ (n = 13/16)	13% (n = 2/15)	40% (n = 6/15)	25% (n = 3/12)
**Low-risk *E6/E7* positive**	28% (n = 5/18)	7% (n = 1/15)	-	-
**High- and low-risk *E6/E7* positive**	28% (n = 5/18)	-	-	-

PET, preeclampsia; FGR, fetal growth restriction; not investigated. ^1^, *p* = 0.0006 compared to the HPV-positive control cohort. -, not tested.

**Table 3:** High- and low-risk HPV *E6*/*E7* in placenta cohorts from Japan.

	HDP (n = 15)	Control (n = 11)
**High-risk HPV DNA positive**	47% (n = 7/15)	9% (n = 1/11)
**High-risk *E6/E7/*high-risk HPV DNA positive**	86% ^1^ (n = 6/7)	0% (n = 0/1)
**Low-risk *E6/E7* positive**	33% ^2^ (n = 5/15)	0%
**High- and low-risk *E6/E7* positive**	13% (n = 2/15)	0%

*HDP, pregnancy-induced hypertension; ^1^ *p* = 0.017; ^2^ *p* = 0.033 compared to the control group.

**Table 4:** High-risk *E6*/*E7* in first-trimester placental tissue.

	Decidua Basalis: Endometrial Epithelium	Decidua Basalis: Stromal Cells/Trophoblast	Chorionic Villi: Trophoblast	Chorionic Villi: Stromal Cells
**High-risk HPV DNA positive**	44% (n = 14/32)	38% (n = 12/32)	22% (n = 7/32)	9% (n = 3/32)
**High-risk *E6*/*E7* RNA positive**	25% (n = 8/32)	22% (n = 7/32)	9% (n = 3/32)	3% (n = 1/32)
**High-risk *E6*/*E7 RNA*/HPV high-risk DNA positive**	57% (n = 8/14)	58% (n = 7/12)	43% (n = 3/7)	33% (n = 1/3)

## Supplementary Materials

The following are available online at https://www.mdpi.com/2076-0817/9/3/239/s1, Figure S1: Assessment of RNA quality in placental tissue sections, Figure S2: Examples of HPV testing in the placenta and HeLa cell control, Figure S3: HPV and p53 staining in the decidua basalis in the placenta, Figure S4: High-risk HPV *E6/E7* expression in the decidua basalis endometrium and trophoblasts in the first-trimester placenta, Table S1: Summary of HPV results in this study.
