# Supplementary material for: Human Papillomavirus E6/E7 Expression in Preeclampsia-Affected Placentae
Source: Pathogens. 2020 Mar 23;9(3):239. doi: 10.3390/pathogens9030239 (PMC7157573; doi:10.3390/pathogens9030239)
Supplement: Supplementary file 1 [file pathogens-09-00239-s001.pdf]

pathogens

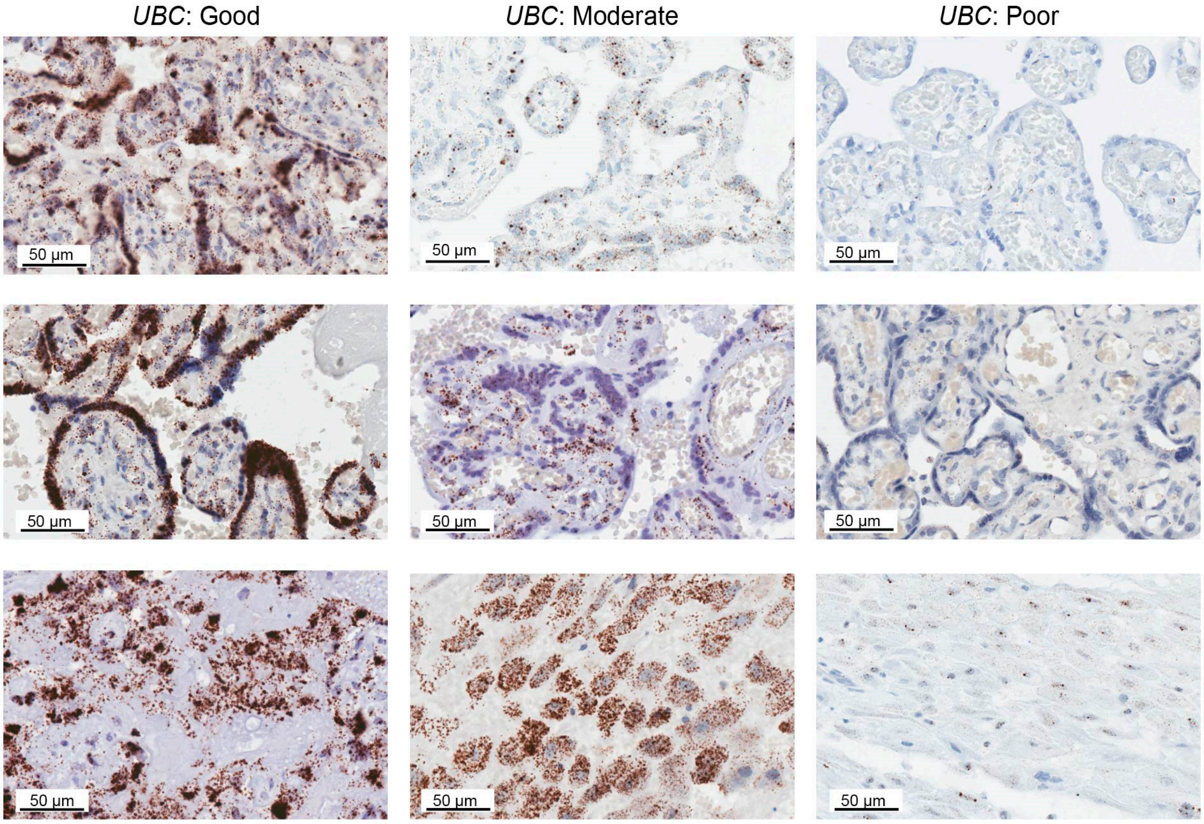

Figure S1: Assessment of RNA quality in placental tissue sections. The RNA quality in placental tissue sections was assessed using a positive control RNAscope assay towards *ubiquitin C* (*UBC*). Cases with good-quality RNA showed strong staining with multiple dots per cell, and almost all cell types were positive. Cases with moderate-quality RNA displayed >10 dots per cell, and most cells were positive. Cases with poor-quality RNA showed patchy or no positive staining regarding cell type or location, or most cells displayed <6 dots per cell. Images shown are from separate cases of placentae collected in New Zealand complicated with pre-eclampsia. Top and middle panels, chorionic villus; bottom panel, decidua basalis.

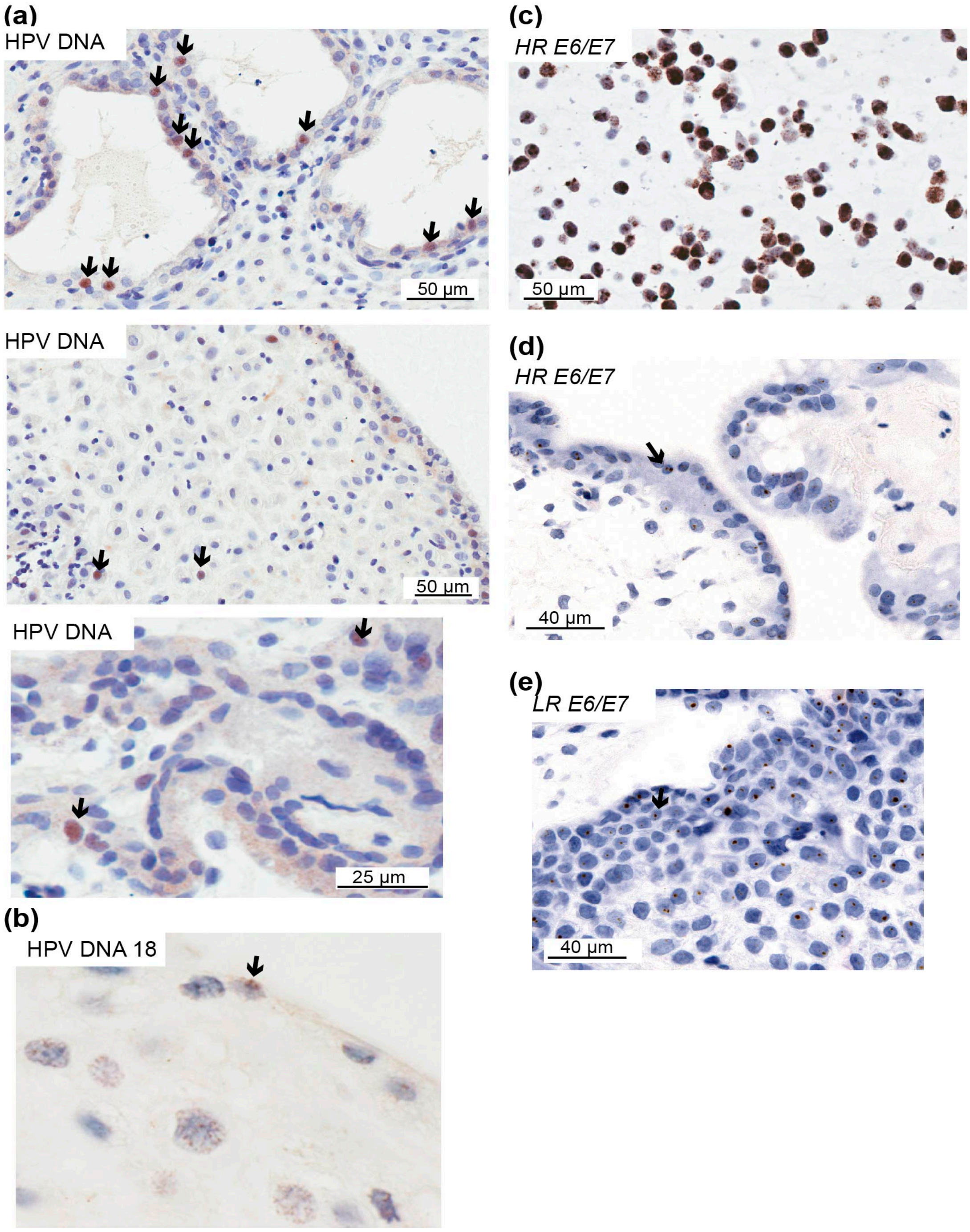

Figure S2: Examples of HPV testing in the placenta and HeLa cell control. **(a)** Placental tissues were assayed for HPV DNA using *in situ* hybridization with a probe towards five high-risk types (16, 18, 31, 33, and 51). HPV was present in the endometrium epithelium and other cell types in the decidua basalis and in the chorionic villi. Examples of positively stained cells are highlighted with arrows. **(b)** Placental tissues assayed for HPV DNA using *in situ* hybridization with a probe towards HPV 18. Positive staining in the decidua basalis is shown. **C.** High-risk (HR) *E6*/*E7* detected using RNAscope was abundantly expressed in HeLa cells. **D.** High-risk *E6*/*E7* expression detected using RNAscope in the syncytiotrophoblast and cytotrophoblast. **E.** Low-risk (LR) *E6*/*E7* expression detected using RNAscope in the anchoring trophoblast. Positively stained cells are highlighted with arrows.

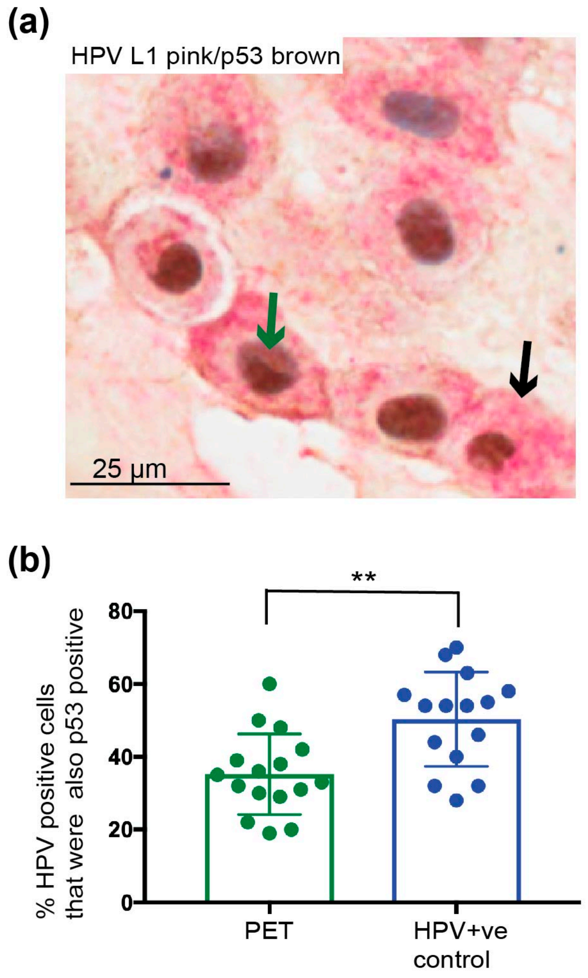

Figure S3: HPV and p53 staining in the decidua basalis in the placenta. Double-labeling immunohistochemistry was used to determine the amount of HPV-positive cells that were also p53 positive. Placentae from the PET and HPV-positive control cohorts from New Zealand were stained for the HPV L1 protein (pink) and p53 (brown). **(a)** An example of HPV L1 (black arrow) and p53 positive (green arrow) staining. **(b)** Comparison of the percentage of HPV-positive cells in the decidua basalis that were also positive for p53 between the PET and HPV-positive control cohorts. ** $p = 0.0016$.

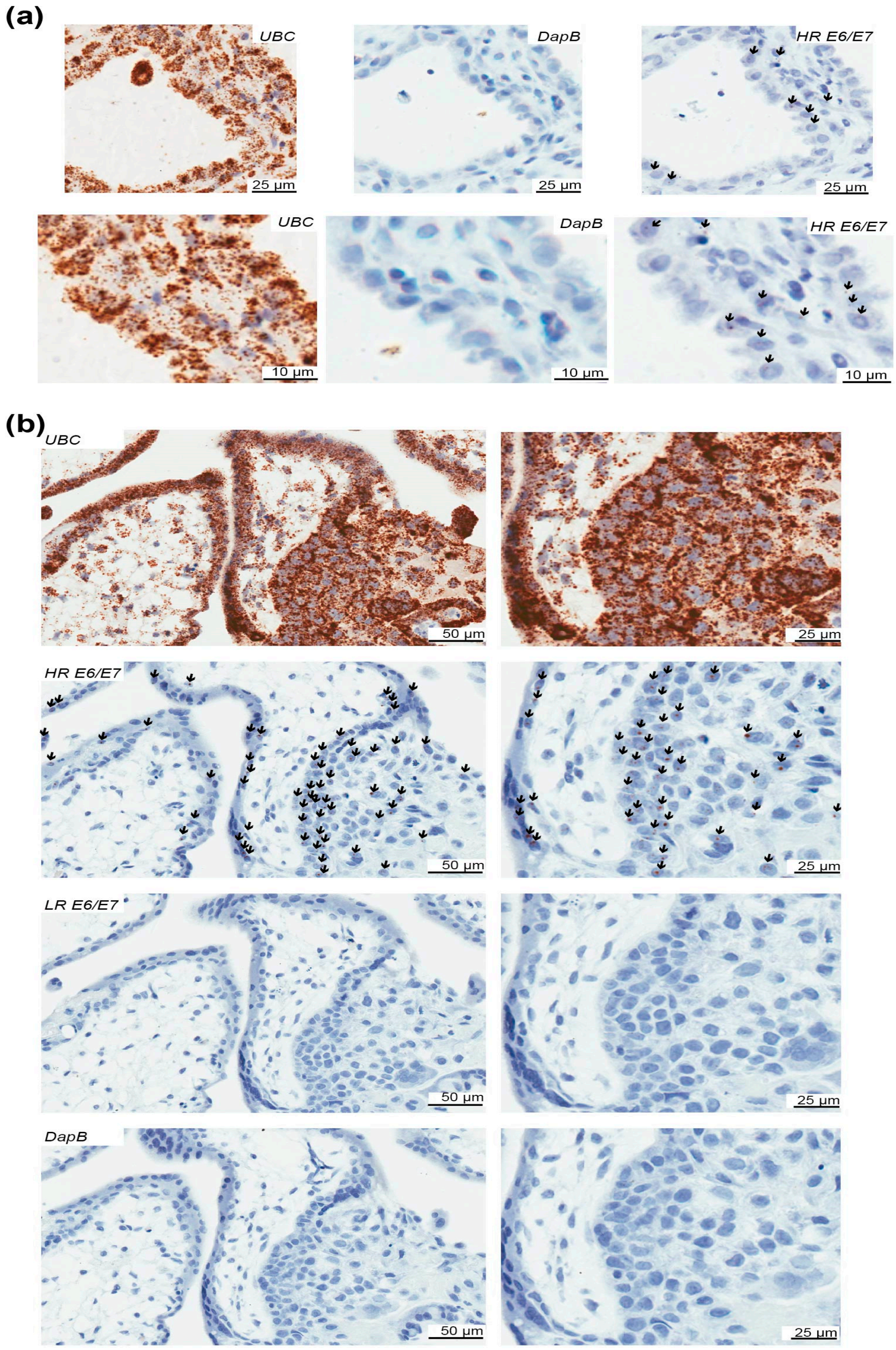

Figure S4: High-risk HPV *E6*/*E7* expression in the decidua basalis endometrium and trophoblasts in the first-trimester placenta. **(a)** The decidua basalis was assayed for HPV gene expression using RNAscope. *Ubiquitin C* (*UBC*) as a positive control for RNA quantity, bacterial gene *DapB* as a negative control, and HPV early genes *E6*/*E7* from 18 high-risk HPV types are shown. Bottom panel, enlarged images taken at the same magnification are shown on the right of the corresponding panel to better illustrate stained cells. Positively stained cells are highlighted with arrows. **(b)** The chorionic villus region was assayed for HPV gene expression using RNAscope. Top panel, *ubiquitin C* (*UBC*) as a positive control for RNA quantity; second panel, early gene expression of *E6*/*E7* from 18 high-risk (HR) HPV types; third panel, *E6*/*E7* gene expression from 10 low-risk (LR) HPV types; and bottom panel, the bacterial gene *DapB* as a negative control. Enlarged images taken at the same magnification are shown on the right of the corresponding panel to better illustrate stained cells. Positively stained cells are highlighted with arrows.

Supplementary Figure 1. Summary of HPV results in this study

**Sample ID**	**High-risk HPV DNA *in situ***	**HPV types**	**High-risk E6/E7 expression**	**Low-risk E6/E7 expression**
**New Zealand cohorts**				
C 1	positive	18 and 26	Negative	Negative
C 2	positive	18	Negative	Negative
C 3	positive	18	Negative	Negative
C 4	Positive	33	Negative	Negative
C 5	Positive	35	Negative	Negative
C 6	Positive	16	Negative	Negative
C 7	Positive	18	Positive	Positive
C 8	Positive	18 and 42	Positive	Positive
C 9	Positive	negative	Negative	Negative
C 10	Positive	negative	Negative	Negative
C 11	Positive	negative	Negative	Negative
C 12	Positive	negative	Negative	Negative
C 13	Positive	negative	Negative	Negative
C 14	Positive	negative	Negative	Negative
C 15	Positive	negative	Negative	Negative
DM 1	Positive	45	Negative	not tested
DM 2	Positive	18 and 40	Negative	not tested
DM 3	Positive	16, 40 and 61	Positive	not tested
DM 4	Positive	18	Negative	not tested
DM 5	Positive	16	Negative	not tested
DM 6	Positive	18 and 42	Positive	not tested
DM 7	Positive	18	Negative	not tested
DM 8	Positive	negative	Negative	not tested
DM 9	Positive	negative	Negative	not tested
DM 10	Positive	negative	Negative	not tested
DM 11	Positive	negative	Positive	not tested
DM 12	Positive	negative	Negative	not tested
FGR 1	Positive	18 and 52	Positive	not tested
FGR 2	Positive	18 and 89	Positive	not tested
FGR 3	Positive	33	Negative	not tested
FGR 4	Positive	16 and 18	Negative	not tested
FGR 5	Positive	18, 52, and 53	Positive	not tested
FGR 6	Positive	18	Negative	not tested
FGR 7	Positive	18 and 26	Negative	not tested
FGR 8	Positive	16	Negative	not tested
FGR 9	Positive	66*	Positive	not tested

FGR 10	Positive	negative	Negative	not tested
FGR 11	Positive	negative	Negative	not tested
FGR 12	Positive	negative	Negative	not tested
FGR 13	Positive	negative	Negative	not tested
FGR 14	Positive	negative	Negative	not tested
FGR 15	Positive	negative	Negative	not tested
PET 1	Positive	18	Positive	Negative
PET 2	Positive	16 and 18	Positive	Negative
PET 3	Positive	18	Positive	Negative
PET 4	Positive	16 and 42	positive	positive
PET 5	Positive	18 and 52	Positive	Negative
PET 6	Positive	66*	Positive	Negative
PET 7	Positive	18 and 52	Positive	Negative
PET 8	Positive	18 and 42	positive	positive
PET 9	Positive	16, 18 and 89	Positive	Negative
PET 10	Positive	45*	Negative	Negative
PET 11	Positive	39*	Positive	Negative
PET 12	Positive	Negative	positive	positive
PET 13	Positive	Negative	Positive	Negative
PET 14	Positive	Negative	Negative	Negative
PET 15	Positive	Negative	Positive	Positive
PET 16	Positive	Negative	Negative	Negative
PET 17	Negative	Negative	Negative	Negative
PET 18	Negative	Negative	Negative	Negative
**Japanese cohorts**				
C 1	Positive	not tested	Negative	Negative
C 1	Negative	not tested	Negative	Negative
C 2	Negative	not tested	Negative	Negative
C 3	Negative	not tested	Negative	Negative
C 4	Negative	not tested	Negative	Negative
C 5	Negative	not tested	Negative	Negative
C 6	Negative	not tested	Negative	Negative
C 7	Negative	not tested	Negative	Negative
C 8	Negative	not tested	Negative	Negative
C 9	Negative	not tested	Negative	Negative
C 10	Negative	not tested	Negative	Negative
C 11	Negative	not tested	Negative	Negative
HDP 1	Positive	not tested	Positive	Negative
HDP 2	Positive	not tested	Positive	Negative
HDP 3	Positive	not tested	Positive	Positive
HDP 4	Positive	not tested	positive	Negative
HDP 5	Positive	not tested	Positive	Negative

HDP 6	Positive	not tested	Positive	Positive
HDP 7	Positive	not tested	Negative	Negative
HDP 8	Negative	not tested	negative	positive
HDP 9	Negative	not tested	Negative	Positive
HDP 10	Negative	not tested	Negative	Positive
HDP 11	Negative	not tested	Negative	Negative
HDP 12	Negative	not tested	negative	Negative
HDP 13	Negative	not tested	Negative	Negative
HDP 14	Negative	not tested	Negative	Negative
HDP 15	Negative	not tested	Negative	Negative

C, NZ high-risk HPV control cohort; DM, diabetic cohort; FGR, fetal growth restriction cohort; PET, preeclampsia cohort. *, the PET and FGR cohorts were included in the Leica ISH staining to HPV DNA and therefore these are cases likely to contain an additional HPV type based on the ISH result (positive for the Lecia HPV probe towards 16, 18, 31, 33 and 51 types).
